# Effect of Mn Deficiency on Carbon and Nitrogen Metabolism of Different Genotypes Seedlings in Maize (*Zea mays* L.)

**DOI:** 10.3390/plants12061407

**Published:** 2023-03-22

**Authors:** Yuzhao Tao, Changzhuang Liu, Lin Piao, Fuqiang Yang, Jiaqi Liu, Muhammad Faheem Jan, Ming Li

**Affiliations:** 1 College of Agriculture, Northeast Agricultural University, Harbin 150030, China; taoyuzhao111@163.com (Y.T.);; 2 Maize Research Institute, Heilongjiang Academy of Agricultural Sciences, Harbin 150086, China

**Keywords:** genotype, hydroponics, manganese, maize, nitrate reductase, photosynthetic rate, sucrose phosphate synthase

## Abstract

Manganese deficiency critically impairs the function and stability of photosystem II (PSII) and negatively impacts crop growth and yield. However, the response mechanisms of carbon and nitrogen metabolism to Mn deficiency in different genotypes of maize and the differences in Mn deficiency tolerance are unclear. Herein, three different genotypes of maize seedlings (sensitive genotype: Mo17, tolerant genotype: B73, and B73 × Mo17) were exposed to Mn deficiency treatment for 16 days using liquid culture with different concentrations of MnSO_4_ [0.00, 2.23, 11.65, and 22.30 mg/L (control)]. We found that complete Mn deficiency significantly reduced maize seedling biomass; negatively affected the photosynthetic and chlorophyll fluorescence parameters; and depressed nitrate reductase, glutamine synthetase, and glutamate synthase activity. This resulted in reduced leaf and root nitrogen uptake, with Mo17 being most severely inhibited. B73 and B73 × Mo17 maintained higher sucrose phosphate synthase and sucrose synthase activities and lower neutral convertase activity compared to Mo17, which resulted in higher accumulation of soluble sugars and sucrose and maintenance of the osmoregulation capacity of leaves, which helped mitigate damage caused by Mn deficiency. The findings revealed the physiological regulation mechanism of carbon and nitrogen metabolism in different genotypes of maize seedlings that resist Mn deficiency stress, providing a theoretical basis for developing high yield and quality.

## 1. Introduction

Manganese (Mn) is an essential micronutrient for the normal growth and development of higher plants [1]. As an important component of the oxygen evolution complex (OEC) of Photosystem II (PSII) [2,3], it strongly influences photosynthetic water oxidation processes [4], carbohydrate metabolism [5], and nitrate assimilation in plants [6].

Carbon metabolism is the most basic physiological metabolic process in plants [7]. The function and stability of plant PSII will be affected by even a mild Mn deficiency [8,9], resulting in impaired electron transfer, significantly reduced photosynthetic efficiency [10,11,12], severely affected photosynthetic carbon fixation and transport, reduced photosynthetic carbon assimilation, impaired crop growth, and reduced yields [13,14]. A fluorescence induction curve showed two new inflection points in OJIP fluorescence transients, i.e., K step and D dip, which indicated that the OEC was severely impaired following Mn deficiency [15]. Moreover, the efficient genotypes maintained a higher maximum quantum yield of PSII (Fv/Fm), net photosynthetic rate (Pn), and leaf Mn concentration. They also accumulated more dry mass per unit of PSII than the inefficient genotypes during Mn deficiency [10,12,16]. Mn deficiency affects the synthesis and transport of carbohydrates. The increase in soluble sugar content during Mn deficiency provides osmoregulatory functions in the root systems of maize, mulberry, and lettuce, thereby stimulating the plant to activate antioxidant defense mechanisms [17,18,19]. Studies on chickpea and mulberry plants demonstrated that the content of reducing sugars in leaves increased and that of starch decreased significantly during Mn deficiency, but nonreducing sugar content changes were inconsistent [18,20]. Sucrose phosphate synthase (SPS) is the key enzyme necessary for sucrose to enter the metabolic pathway, with its activity affecting sucrose biosynthesis ability. Sucrose synthase (SS) is responsible for the reversible reactions of sucrose breakdown and synthesis [21]. Neutral convertase (NI) mainly catalyzes the conversion of sucrose to glucose and fructose, and these three enzymes strongly influence sucrose metabolism [22]. Nevertheless, little is known about the effect of Mn deficiency on the activity of key sugar metabolism enzymes in plants, and there remains controversy regarding nonstructural carbohydrate synthesis and transport.

Mn stress negatively affects the nitrogen metabolic system in plants [23]. Changes in the Mn^2+^ concentration influences the utilization of nitrate or ammonium as a nitrogen source in plants [24], and Mn deficiency is not conducive to nitrate assimilation. Nitrate reductase (NR) is a highly critical rate-limiting enzyme in plant nitrogen metabolism, with the magnitude of activity reflecting the ability of plants to absorb and utilize nitrogen. Glutamine synthetase (GS) and glutamate synthase (GOGAT) together create the GS–GOGAT cycle, which is the main pathway for plant ammonia assimilation [7]. Glutamate dehydrogenase (GDH) catalyzes the synthesis of α-ketoglutarate from glutamate, which in turn provides the carbon skeleton necessary for amino acid synthesis [22]. Gong et al. found that Mn deficiency inhibited nitrate nitrogen uptake and transport in maize seedling leaves, while NR and GS activity decreased, and GDH activity increased [25]. The total nitrogen and protein contents were reduced in chickpea, sugarcane, maize, and tobacco under Mn deficiency [11,20,25,26]. Although Mn-efficient genotypes maintain higher NR activity than less efficient genotypes during Mn deficiency in rice [16], little is known about whether there are differences between nitrogenous compounds and nitrogen assimilation-related enzyme activities of different tolerant genotypes during Mn deficiency. Nitrogen is an important mineral nutrient in plant growth and development, and the root system is the main organ used to absorb nitrogen [27]. Plant root growth decreased, as did biomass, during Mn deficiency [19,28], but how this deficiency influences nitrogen metabolism in plant roots remains unclear.

Mn-deficient soils are widely distributed, and approximately 30% of the soils in China are impacted by Mn deficiency [29]. Maize is one of the most essential food crops globally, and Mn deficiency in the early growth stages results in significant suppression of physiological functions in maize. Previously, Fv/Fm has represented an important indicator for selecting low Mn tolerance genotypes in plants, and maize seedlings of tolerant genotypes may maintain optimal Fv/Fm during longer periods of Mn deficiency stress compared to sensitive genotypes [8]. In a previous study, B73 was the low-Mn tolerant genotype and Mo17 was the low-Mn sensitive genotype, and there were significant differences in dry weight between different tolerant genotypes; these differences were detrimental to yield production [10]. However, the effects of Mn deficiency on carbon and nitrogen metabolism in maize seedlings of different genotypes remain poorly understood.

This study aimed to investigate the effects of Mn deficiency on the carbon and nitrogen metabolism of maize seedlings of different tolerant genotypes and the response differences between genotypes. This was accomplished by measuring photosynthesis and chlorophyll fluorescence parameters and enzyme activities related to carbon and nitrogen metabolism, nonstructural carbohydrate content, and nitrogen compounds content under Mn deficiency conditions, and by further exploring the differences in Mn deficiency tolerance among different varieties. The findings provide a theoretical basis for high yield and quality of maize.

## 2. Results

### 2.1. Effect of Mn Deficiency on the Growth of Maize Seedlings of Different Genotypes

#### 2.1.1. Effect of Mn Deficiency on Leaf Dry Weight and Leaf Phenotype

**Figure 1:**
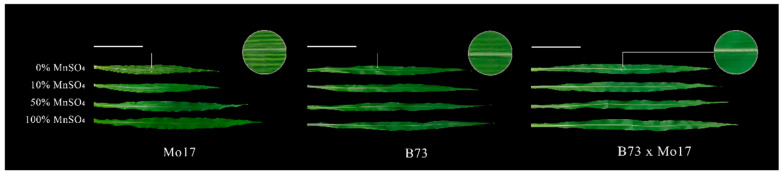
Effect of Mn deficiency on the leaves of maize seedlings, scale bar = 10 cm.

In a two-way ANOVA (Table 1), it was found that genotypes, MnSO_4_ concentration, and genotypes × MnSO_4_ concentration interactions had highly significant effects on shoot dry weight, root dry weight, and plant height of maize seedlings (*p* < 0.01), except for the genotype × MnSO_4_ concentration interaction, which had no significant effect on root dry weight (*p* > 0.05). Sensitive genotype Mo17 showed a streak-like green loss during Mn deficiency stress, but the tolerant genotype seedlings did not show obvious green loss symptoms regardless of stress severity (Figure 1, Table 2). Mn deficiency severely affected the shoot and root dry weight accumulation, plant height, and leaf area of the seedlings, which all showed strong negative correlations with Mn deficiency. These correlations were more evident in the sensitive genotype Mo17 than in the tolerant genotype B73. During a complete Mn deficiency treatment, the shoot and root dry weights, plant height, and leaf area of Mo17 and B73 decreased by 51.19%, 27.46%, 28.53%, and 37.63% (*p* < 0.05) and by 20.75%, 19.56%, 12.87%, and 26.34% (*p* < 0.05), respectively. The root to shoot ratio (R/S) of Mo17 showed a positive correlation with Mn deficiency, and during complete Mn deficiency, it increased significantly by 49.17% (*p* < 0.05). However, there were no significant changes in R/S of B73. The shoot and root dry weights, the plant height, and leaf area of the hybrid B73 × Mo17 were significantly higher than those of the parental self-crosses, showing a significant superparental advantage. The R/S was similar to that of B73, and no significant changes were observed under Mn deficiency.

#### 2.1.2. Effect of Mn Deficiency on the Root System of Maize Seedlings

**Figure 2:**
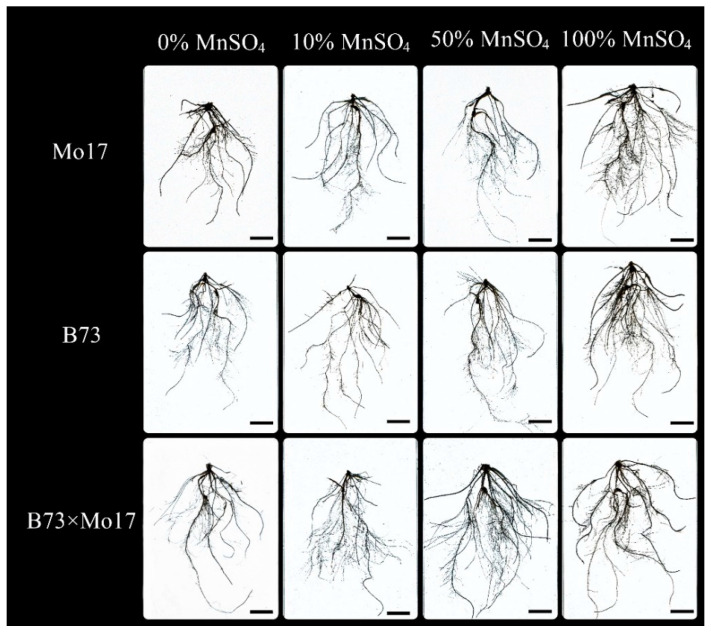
Effect of Mn deficiency on root morphology of maize seedlings, scale bar = 5 cm.

The two-way ANOVA (Table 3) showed that genotypes, MnSO_4_ concentration, and genotypes × MnSO_4_ concentration interactions had a highly significant effect on total root length of maize seedlings (*p* < 0.01). The total root length, root surface area, root volume, and root vigor of maize seedlings were significantly inhibited during Mn deficiency stress (Figure 2, Table 4). Under 50% MnSO_4_ concentration treatment, the above indicators of sensitive genotype Mo17 significantly decreased by 55.53%, 51.63%, 35.59%, and 32.65%, respectively (*p* < 0.05), while the same indicators of B73 showed no significant changes. With complete Mn deficiency, the same indicators of Mo17 and B73 decreased by 71.44%, 71.78%, 77.31%, and 50.30% (*p* < 0.05) and 30.23%, 34.34%, 42.34%, and 42.11% (*p* < 0.05), respectively; Mo17 showed clear impairment. The average root diameter of B73 decreased significantly under Mn deficiency stress (*p* < 0.05), but the average root diameter of Mo17 showed no significant variations. The total root length, root surface area, and root volume of the hybrid B73 × Mo17 were all larger than the parental self-incompatible lines and maintained higher levels under low Mn conditions.

### 2.2. Effect of Mn Deficiency on Photosynthesis and Chlorophyll Fluorescence in Maize Seedlings of Different Genotypes

#### 2.2.1. Effects of Manganese Deficiency on Chlorophyll Content and Photosynthesis in Maize Seedlings

**Figure 3:**
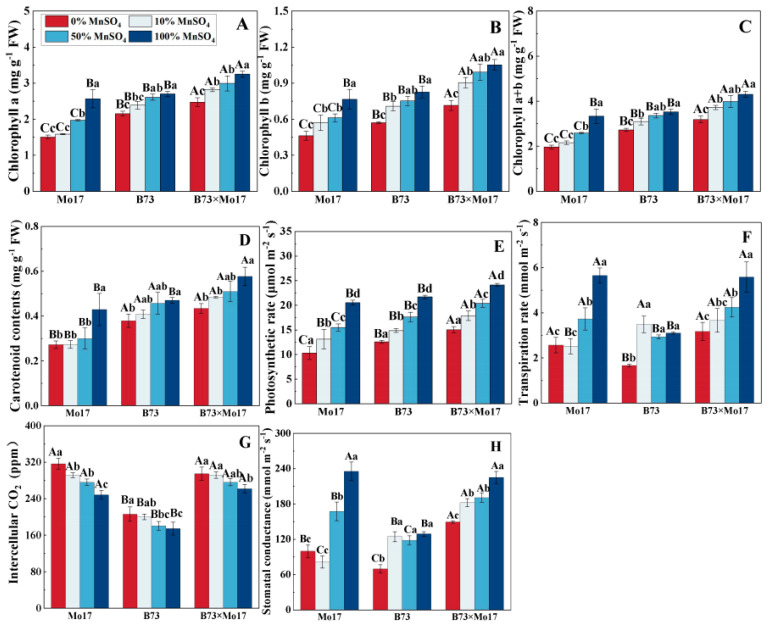
Effect of Mn deficiency on photosynthetic pigment contents and photosynthetic gas exchange parameters of maize seedling leaves. Chlorophyll a (**A**), chlorophyll b (**B**), chlorophyll a+b (**C**), carotenoid contents (**D**), photosynthetic rate (**E**), transpiration rate (**F**), intercellular CO_2_ (**G**), stomatal conductance (**H**). According to the LSD test, different capital letters: significant difference between varieties (*p* < 0.05); different lowercase letters: significant difference between MnSO_4_ treatments (*p* < 0.05). Data are expressed as the mean of three replicates.

Mn deficiency resulted in a marked decrease in chlorophyll and carotenoid content of maize seedlings (Figure 3), with the extent of decrease being Mo17 > B73 > B73 × Mo17. The photosynthetic pigment content of Mo17 decreased significantly (*p* < 0.05) with a 50% Mn concentration treatment, but there were no significant changes in B73. During complete Mn deficiency, chlorophyll a, chlorophyll b, chlorophyll a+b, and carotenoid contents of the Mo17 and B73 maize seedlings decreased by 41.41%, 39.56%, 40.99%, and 36.60% (*p* < 0.05) and 20.24%, 30.89%, 22.73%, and 19.41% (*p* < 0.05), respectively, and the above indicators of hybrids B73 × Mo17 decreased by 23.34%, 32.08%, 25.86%, and 24.80% (*p* < 0.05), respectively. The photosynthetic pigment contents of B73 × Mo17 were significantly higher than the parental self-crosses during Mn deficiency, reaching superparental dominance.

The net photosynthetic rate (Pn), transpiration rate (Tr), and stomatal conductance (Gs) of maize seedling leaves significantly decreased, and the intercellular CO_2_ concentration (Ci) increased during Mn deficiency, with the greatest variations occurring in Mo17. During mild Mn deficiency, Mo17 was the first to demonstrate signs of Mn deficiency stress, and the changes in indicators all reached the significance level. Pn, E, and Gs of Mo17 and B73 maize seedlings decreased by 49.93%, 54.55%, and 57.47% (*p* < 0.05) and 42.07%, 46.35%, and 45.89% (*p* < 0.05), respectively, when completely deprived of Mn, while the Ci increased by 27.52% and 18.31%, respectively. Additionally, B73 significantly decreased in Tr and Gs only during complete Mn deficiency. The Pn, Tr, and Gs of the hybrid B73 × Mo17 reached superparental levels under Mn deficiency treatment.

#### 2.2.2. Effect of Mn Deficiency on Chlorophyll Fluorescence in Maize Seedlings

**Figure 4:**
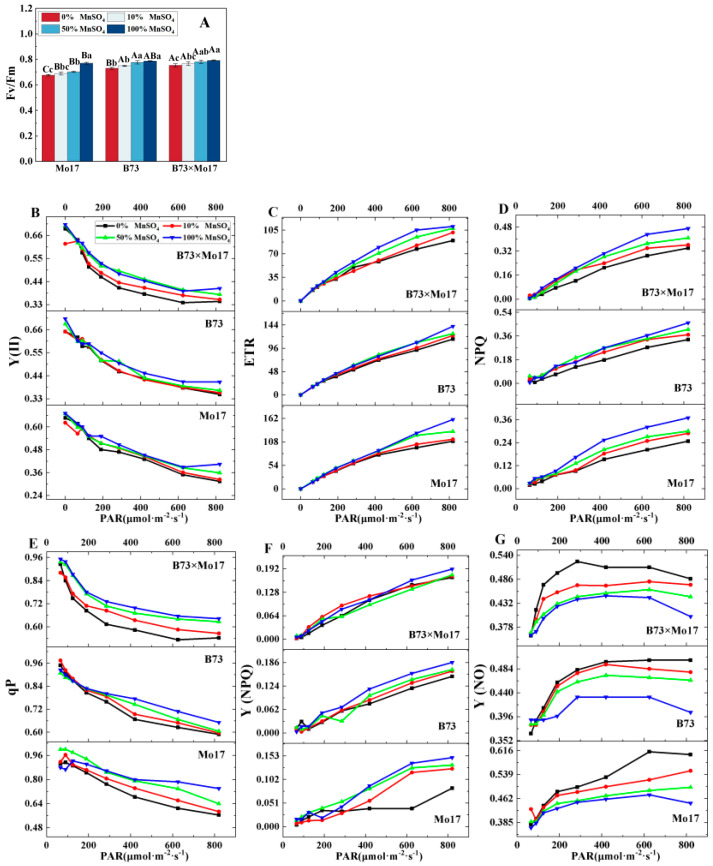
Effect of Mn deficiency on chlorophyll fluorescence parameters of maize seedling leaves. Fv/Fm: maximum photochemical efficiency (**A**), Y(II): photochemical quantum yield of PSII (**B**), ETR: electron transport rate (**C**), NPQ: Non-photochemical fluorescence quenching (**D**), qP: photo-chemical quenching coefficient (**E**), Y(NPQ): quantum yield of non-photochemical fluorescence quenching in light-acclimated samples (**F**), Y(NO) quantum yield of non-photochemical fluoresc-ence quenching in dark-acclimated samples (**G**). According to the LSD test different capital letters: significant difference between varieties (*p* < 0.05); different lowercase letters: significant difference between MnSO_4_ treatments (*p* < 0.05). Data are expressed as the mean of three replicates.

Changes in photosynthetic fluorescence parameters can respond to the inherent characteristics of photosynthesis when plants are stressed. Figure 4 shows that Fv/Fm, Y(II), ETR, NPQ, qP, and Y(NPQ) decreased significantly, and Y(NO) increased in maize seedling leaves during Mn deficiency. Mo17 responded most strongly to complete Mn deficiency; the same parameters decreased by 12.13%, 22.28%, 31.29%, 32.97%, 23.78%, and 44.30%, (*p* < 0.05), respectively, while the Ci increased by 34.90%. B73 was relatively less affected, and the parameters decreased by 7.16%, 14.39%, 18.63%, 27.61%, 9.52%, and 19.89% (*p* < 0.05), respectively, and Ci increased by 23.76%. The above indicators of hybrid B73 × Mo17 decreased by 4.96%, 15.23%, 18.91%, 27.66%, 15.58%, 12.04%, and −21.14% (*p* < 0.05), respectively. The trend of the hybrid B73 × Mo17 indicators was similar to that of B73 during Mn deficiency, and B73 × Mo17 had a strong tolerance to the Mn-deficient environment; the Fv/Fm was higher than that of the parental self-crosses, which had a superparental advantage.

### 2.3. Effect of Mn Deficiency on Carbon Metabolism in Maize Seedlings of Different Genotypes

**Figure 5:**
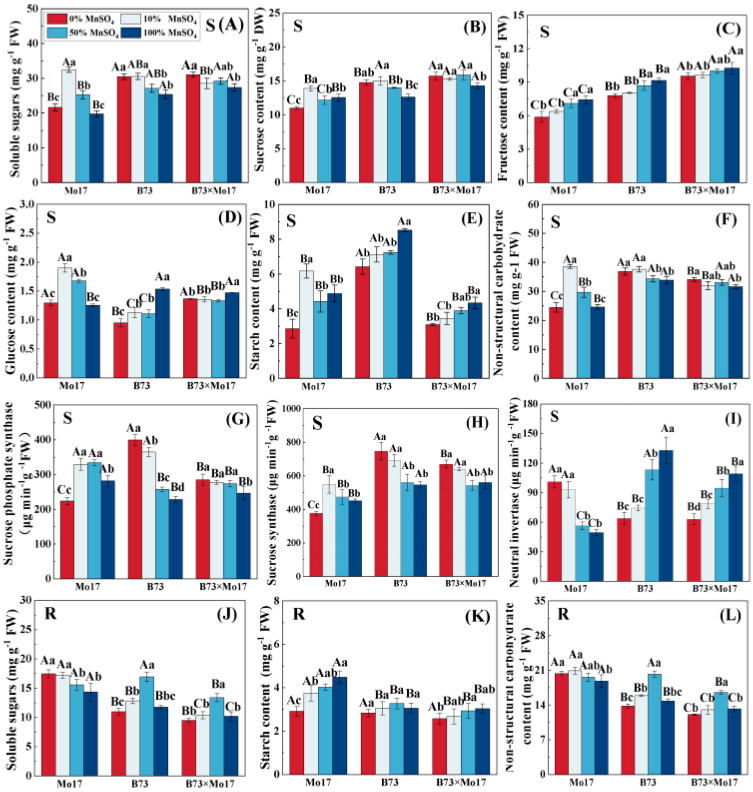
Effect of Mn deficiency on soluble carbohydrate content and carbon metabolism-related enzyme activities in maize seedlings. Soluble sugars (**A**,**J**), sucrose content (**B**), fructose content (**C**), glucose content (**D**), starch content (**E**,**K**), nonstructural carbohydrate (**F**,**L**), sucrose phosphate syn-thase (**G**), sucrose synthase (**H**), neutral invertase (**I**). According to the LSD test different capital letters: significant difference between varieties (*p* < 0.05); different lowercase letters: significant dif-ference between MnSO_4_ treatments (*p* < 0.05). S represents leaf-related parameters, R indicates root-related parameters. Data are expressed as the mean of three replicates.

The changes in soluble carbohydrate content and carbon metabolism enzyme activities of maize seedlings of different genotypes showed considerable variation during Mn deficiency. The soluble sugars, sucrose, glucose, starch, NSC, SPS, and SS (synthesis direction) activities of Mo17 leaves increased, then decreased, with an increase in Mn deficiency. The above indicators of Mo17 reached a peak at 10% Mn concentration and demonstrated a decreasing trend with a further increase in Mn deficiency (Figure 5). Among them, sucrose, starch, SPS, and SS activities were significantly lower than the control level (100% MnSO_4_) and decreased by 12.39%, 41.48%, 20.61%, and 16.58%, respectively, when compared to the control during complete Mn deficiency (*p* < 0.05). The soluble sugar and glucose contents as well as NSC decreased by 33.44%, 31.72%, and 36.68% (*p* < 0.05), respectively, when compared to 10% MnSO_4_ treatment in complete Mn deficiency, but no significant differences were observed compared to the control. Furthermore, the leaf fructose content showed a linear correlation with Mn concentration: the lower the Mn concentration, the lower the leaf fructose. However, Mn deficiency resulted in a significant increase in NI. Leaf NI activity was 2.04 times higher than the control when there was complete Mn deficiency. When Mn was completely removed, soluble sugars, starch, and NSC in the root system increased by 21.75%, −34.97%, and 8.25%, respectively, compared to that in control (*p* < 0.05).

Unlike in the sensitive genotype, the leaf soluble sugars, sucrose, NSC, and SPS, as well as the SS activities of B73, showed an increasing trend in total Mn deficiency (Figure 5), increasing by 20.05%, 17.19%, 8.8%, 75.18%, and 36.69% (*p* < 0.05), respectively, and reaching the significant level. The leaf NI activity, fructose, glucose, and starch contents decreased significantly with increasing Mn deficiency. During complete Mn deficiency, they were reduced by 52.04%, 14.89%, 37.95%, and 24.66% (*p* < 0.05), respectively. The trends of the root NSC and root soluble sugar content were similar, rising first and then decreasing, and were significantly higher than the control 36.34% and 43.90% (*p* < 0.05) at 50% MnSO_4_ treatment; no significant difference was observed for complete Mn deficiency. No significant changes in root starch content of maize seedlings were observed during Mn deficiency.

The trend of carbon metabolism changes in B73 × Mo17 was similar to that of B73, and the leaves maintained higher soluble sugars, sucrose, fructose, glucose, NSC, SS, and SPS, and lower NI activity during Mn deficiency (Figure 5). Among them, sucrose and fructose were significantly higher than the parental self-inbred lines, reaching the superparental levels. The trends of root soluble sugars, starch, and NSC were similar to those of B73.

### 2.4. Effect of Mn Deficiency on Nitrogen Metabolism in Maize Seedlings of Different Genotypes

#### 2.4.1. Effect of Mn Deficiency on Nitrogen Metabolites in Maize Seedlings

**Figure 6:**
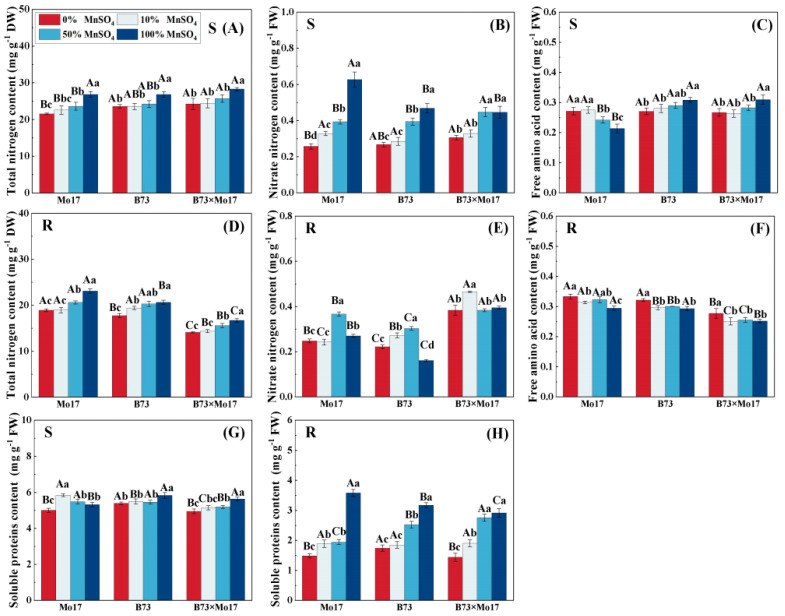
Effect of Mn deficiency on the content of nitrogenous compounds in nitrogen metabolism of maize seedlings. Total nitrogen content (**A**,**D**), nitrate nitrogen content (**B**,**E**), free amino acid con-tent (**C**,**F**), soluble proteins content (**G**,**H**). According to the LSD test different capital letters: signif-icant difference between varieties (*p* < 0.05); different lowercase letters: significant difference be-tween MnSO_4_ treatments (*p* < 0.05). S represents leaf-related parameters, R indicates root-related parameters.

The total nitrogen content of maize seedling leaves and roots decreased significantly during Mn deficiency (Figure 6). The total nitrogen content of Mo17, B73, and hybrid B73 × Mo17 leaves decreased by 19.65%, 12.00%, and 15.64% (*p* < 0.05), respectively, and the root systems’ parameters decreased by 18.18%, 14.12%, and 14.36%, respectively (*p* < 0.05), during total Mn deficiency. During Mn deficiency stress, leaf nitrate-nitrogen content decreased significantly, and the decrease was manifested as Mo17 (58.97%) > B73 (43.00%) > B73 × Mo17 (31.55%) (*p* < 0.05). The nitrate-nitrogen content of the root first increased and then decreased. Moreover, the root soluble protein content of maize seedlings of different genotypes all decreased significantly as Mn decreased. The root soluble protein content of Mo17, B73, and hybrid B73 × Mo17 decreased by 58.48%, 45.01%, and 50.66%, respectively, during complete Mn deficiency (*p* < 0.05). The soluble protein content of Mo17 leaves increased and then decreased; this value peaked with the 10% MnSO_4_ treatment, showing a 9.73% increase compared to the control (*p* < 0.05). It then decreased significantly by 5.94% under complete Mn deficiency (*p* < 0.05). Additionally, the changes in soluble protein content in B73 and B73 × Mo17 leaves were consistent with that in the root system. The free amino acid content of the root system of Mo17 and B73 maize seedlings significantly increased during Mn deficiency stress, at 1.13 and 1.10 times higher than the control when completely deficient in Mn (*p* < 0.05). The free amino acid content of Mo17 leaves was consistent with the root system, at 1.27 times higher than the control when completely deprived of Mn (*p* < 0.05). In contrast, the free amino acid content of B73 leaves was significantly lower, and the hybrid B73 × Mo17 showed similar changes to B73.

#### 2.4.2. Effect of Mn Deficiency on the Activities of Key Enzymes of Nitrogen Metabolism in Maize Seedlings

**Figure 7:**
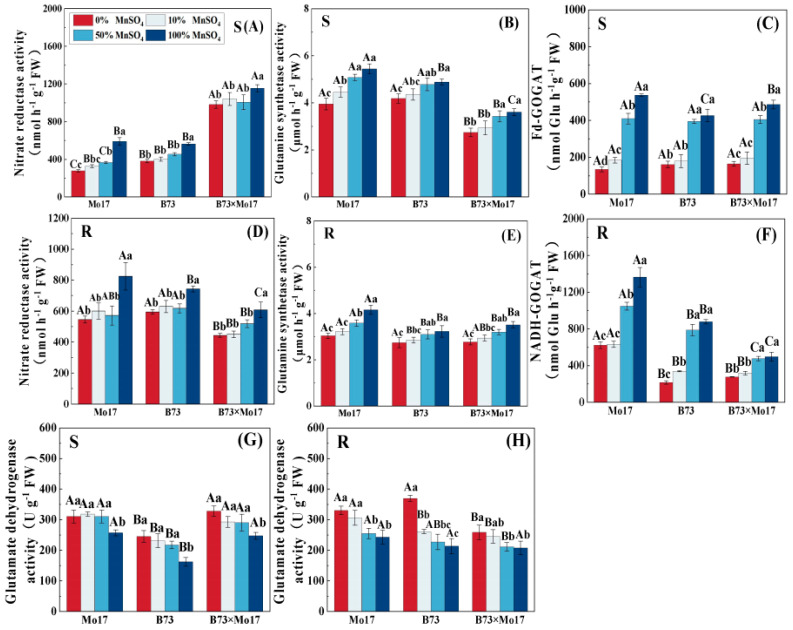
Effect of Mn deficiency on the activities of enzymes in nitrogen metabolism of maize seed-lings. Nitrate reductase activity (**A**,**D**), glutamine synthetase activity (**B**,**E**), Fd-GOGAT (**C**,**F**), gluta-mate dehydrogenase activity (**G**,**H**). According to the LSD test different capital letters: significant difference between varieties (*p* < 0.05); different lowercase letters: significant difference between MnSO_4_ treatments (*p* < 0.05). S represents leaf-related parameters, R indicates root-related parame-ters.

Mn deficiency significantly reduced NR, GS, and GOGAT activities and increased GDH activity in maize seedling leaves and roots. The changes in nitrogen-metabolizing enzyme activity were greater with increasing Mn deficiency (Figure 7), and the tolerant genotypes were less inhibited than the sensitive genotypes. During complete Mn deficiency, Mo17 and B73 leaf NR activity decreased by 52.96% and 32.35% (*p* < 0.05), respectively, and the root system NR activity declined by 33.79% and 19.99% (*p* < 0.05). The B73 leaf and root system GDH activity increased more than that of Mo17. The leaf NR activity of the hybrid B73 × Mo17 was significantly higher than that of the parental self-crosses, reaching superparental levels. Although the root GOGAT was lower than those of its parents were, it was least impacted during complete Mn deficiency.

### 2.5. Correlation Analysis and C/N Ratio

**Figure 8:**
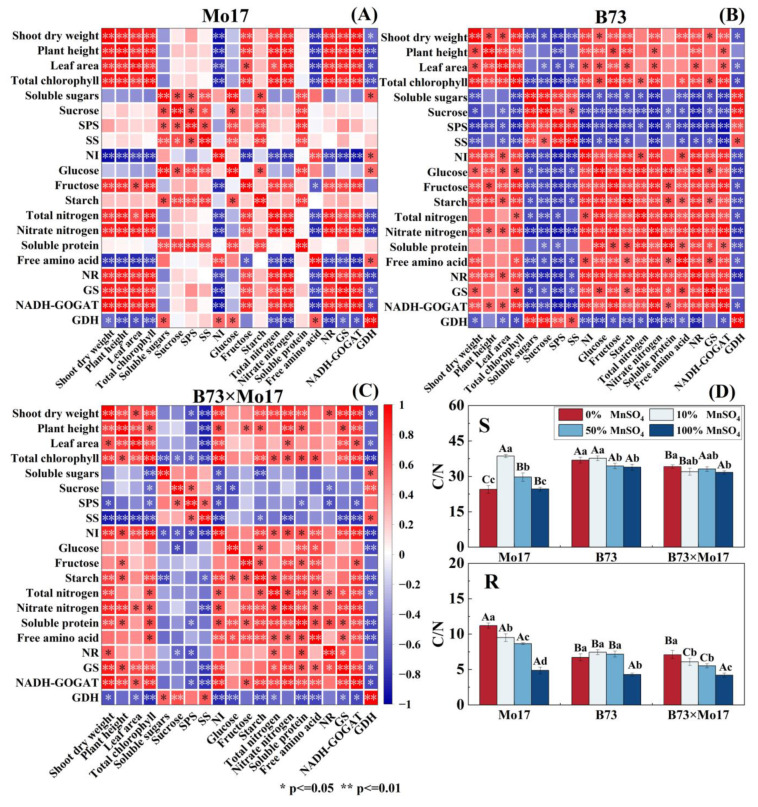
Correlation among dry weight, plant height, leaf area, and carbon and nitrogen metabo-lism in leaves of maize seedlings Mo17 (**A**), B73 (**B**), B73 × Mo17 (**C**) and carbon-to-nitrogen ratio (**D**). According to the LSD test different capital letters: significant difference between varieties (*p* < 0.05); different lowercase letters: significant difference between MnSO_4_ treatments (*p* < 0.05). S rep-resents leaf-related parameters, R indicates root-related parameters.

In sensitive genotypes with Mn deficiency (Figure 8), shoot dry weight, plant height, leaf area, and total chlorophyll content were strongly positively correlated with NR, GS, and GOGAT in leaf nitrogen metabolism; positively correlated with total nitrogen content; positively correlated with fructose content in carbon metabolism except for the leaf area; and negatively correlated with NI. In carbon metabolism, soluble sugars, sucrose, SPS, SS, glucose, and starch were significantly and positively correlated with each other, and the NI activity was strongly negatively correlated with fructose content. In nitrogen metabolism, NR was strongly correlated with total nitrogen, nitrate nitrogen, GS, GOGAT, and GDH. Between carbon and nitrogen metabolism, soluble proteins were closely positively correlated with soluble sugars, sucrose, glucose, starch, and SPS. Overall, maize seedling growth was closely related to nitrogen metabolism enzyme activity, and the correlation between leaf carbon metabolism and nitrogen metabolism was weak.

There was a significant correlation between shoot dry weight, total chlorophyll content, NR, GS, GOGAT, and GDH during Mn deficiency for B73. Moreover, these indicators were significantly positively correlated with starch, fructose, glucose, and NI and negatively correlated with SPS, SS, sucrose, and soluble sugars in carbon metabolism (Figure 8). Among them, NR, GOGAT, SPS, SS, NI, fructose, and starch content showed high correlation levels. In carbon metabolism, there were strongly positive correlations among soluble sugars, sucrose, SPS, and SS and closely negative correlations between sucrose and NI, glucose, fructose, and starch. Notably, there was a strong correlation between carbon and nitrogen metabolism in B73 leaves, with strong negative correlations between leaf soluble sugar, sucrose, SPS and SS activities, and nitrogenous compounds and nitrogen metabolizing enzyme activities; most reached significant or highly significant levels. The hybrid B73 × Mo17 nitrogen metabolism indices were negatively correlated with soluble sugars, sucrose, SS, and SPS of carbon metabolism and positively correlated with glucose, fructose, NI, and GDH, which was a trend similar to that of the tolerant genotype B73.

The C/N ratio of B73 and hybrid B73 × Mo17 leaves increased significantly by 17.83% and 23.11%, respectively, during complete Mn deficiency (*p* < 0.05). The C/N ratio of Mo17 leaves increased first and then decreased, reaching its peak during the 10% MnSO_4_ concentration treatment, and was 41.87% higher than the control. The root C/N of maize seedlings significantly increased during Mn deficiency. The root C/N of Mo17 was elevated by 130.11%. The root C/N of B73 and B73 × Mo17 was elevated by 56.45% and 69.48% (*p* < 0.05), respectively, during 0% MnSO_4_ treatment. The magnitude of variations in leaf and root C/N were greater in the sensitive genotype (Figure 8).

## 3. Discussion

### 3.1. Effect of Mn Deficiency on Carbon Metabolism in Maize Seedlings of Different Genotypes

**Figure 9:**
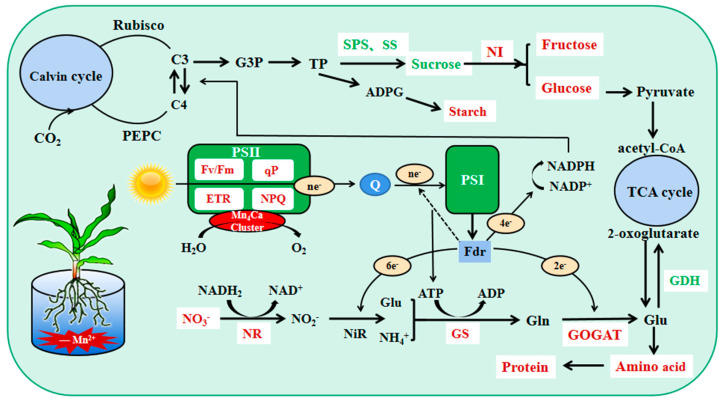
Physiological mechanism of the effect of Mn deficiency on carbon and nitrogen metab-olism in leaves of maize seedlings of tolerant genotype. Red indicates decrease in Mn deficiency, and green indicates increase in Mn deficiency.

Plant carbon metabolism comprises three steps: carbon assimilation and fixation by converting inorganic carbon to organic carbon through photosynthesis, carbohydrate interconversion, and carbon accumulation [7]. Mn_4_CaO_5_ is involved in PSII decomposition of water and the subsequent release of oxygen, which is the first step of photosynthesis [19]. The chloroplast localized Mn transporter protein PAM71 is required for Mn transport into the thylakoid lumen [30]. Mn deficiency mainly affects plant PSII functions, leading to a significant decrease in photosynthetic activity [31]. In this study, we showed that the Pn, Gs, Tr, and photosynthetic pigment contents of maize seedling leaves decreased and Ci increased during Mn deficiency; Fv/Fm, ETR, NPQ, qP, Y(II), and Y(NPQ) decreased significantly, whereas Y(NO) increased significantly. Overall, the tolerant genotype was able to maintain a healthier status compared to the sensitive genotype. The ability of sensitive genotypes of maize seedlings to convert light energy into chemical energy was more severely impaired by Mn deficiency stress, and electron transport on the receptor side of PSII was severely impaired. This probably resulted from the negative effect of Mn deficiency on water photolysis and the impaired function of the electron transport chain on the chlorophyll thylakoid membranes from PSII to PSΙ [32,33]. We also found that the tolerant genotype mitigates damage to the photosynthetic machinery by dissipating excess light energy in the form of heat, whereas the sensitive genotype photochemical energy conversion and protective regulatory mechanisms cannot completely process the absorbed light energy; this leads to increasingly severe photodamage in maize seedlings, which was also reported in previous studies [2,12,34,35]. Moreover, we observed that the young leaves of sensitive genotype maize seedlings showed striped chlorosis when they were slightly manganese deficient, and the leaf area decreased significantly, especially when they were completely manganese-deficient. However, there was no visual striation in the tolerant genotype except during complete manganese deficiency. The photosynthetic capacity of maize seedlings decreased during Mn deficiency, and electron transfer was impaired. This resulted in an inability to provide sufficient reducing power and energy for maize seedling carbon and nitrogen metabolism, which is not conducive to carbohydrate production and nitrogen assimilation (Figure 9). The tolerant genotype maintained a relatively higher photosynthetic capacity.

Soluble carbohydrate content is an important indicator of carbon conversion and accumulation in plants [7], which strongly influences plant resistance to abiotic stresses, provides essential energy and carbon skeletons for plant nitrogen metabolism, and provides essential energy for plant growth and metabolism [36]. Our results indicate that different genotypes of maize seedlings showed significant differences in carbon metabolism during Mn deficiency, with the tolerant genotype maintaining a higher level of carbon metabolism as Mn deficiency stress increased, compared to the sensitive genotype. We suggest that the substantial accumulation of sucrose and soluble sugar contents in the leaves of tolerant genotypes may be a positive feedback regulatory mechanism for their adaptation to stress adversity. It is possible that the increased activity of SPS and SS in leaves during Mn deficiency contributed to the increase in sucrose synthesis. The correlation analysis further demonstrated that both SPS and SS (synthesis direction) activities were significantly and positively correlated with sucrose content (Figure 8); this finding is consistent with those of previous reports [37,38]. A study of cotton metabolism showed that increasing leaf sucrose and total sugar concentrations during Mn stress is a strategy for cotton plants to resist oxidative stress [39]. However, we found that the increase in sucrose synthesis may also be related to the reduction in NI activity. The reduction in NI activity during Mn deficiency stress reduced the translocation of sucrose to glucose and fructose, resulting in a large accumulation of sucrose and a decrease in glucose and fructose contents in leaves. Because ADP-glucose is a direct substrate for starch synthesis [40], the starch content was significantly reduced under Mn deficiency; this dynamic was also likely related to the decrease in the glucose content of the synthetic precursor. However, a previous study in chickpeas suggests that this might have resulted from a decrease in starch phosphorylase activity [20]. Notably, differing from the tolerant genotype, the sensitive genotype showed increasing and then decreasing sucrose and soluble sugar contents in maize seedling leaves during Mn deficiency stress; these contents were significantly lower than the control level during complete Mn deficiency. Maize seedlings showed similar trends in SPS, SS, glucose content, starch content, and NSC, which may be related to the significant reduction of photosynthesis in sensitive genotypes during Mn deficiency stress. During complete Mn deficiency, plants were unable to produce additional soluble carbohydrates by photosynthesis themselves to supply seedling growth. This indicates that the sensitive genotype is weakly resistant to Mn deficiency stress, and its carbon metabolism capacity is significantly suppressed, resulting in a significant reduction in plant biomass. Moreover, NI activity of sensitive genotypes significantly increased during Mn deficiency stress, which likely further contributed to the accumulation of glucose content and indirectly caused changes in starch content. Previous studies have also shown that glucose concentrations are related to NI activity [22].

In conclusion, Mn deficiency significantly reduced the capacity of photosynthesis and the electron transfer rate in maize seedlings, affected the accumulation of soluble carbohydrates in the internal system, and was detrimental to the growth of maize seedlings. Nevertheless, the tolerant genotype can resist Mn deficiency stress by increasing carbon metabolism enzyme activity, accumulating additional soluble carbohydrates to enhance osmoregulation, improving its carbon metabolism level, promoting carbon conversion in maize seedlings, and providing reducing power, energy, and a carbon skeleton for nitrogen metabolism required for growth and development.

### 3.2. Effect of Mn Deficiency on Nitrogen Metabolism in Maize Seedlings of Different Genotypes

Nitrate nitrogen and ammonium nitrogen absorbed by plants from the soil are combined with carbon sources and ATP and converted into amino acids and proteins. Enzymes involved include NR, GS, GOGAT, and GDH, which are essential for the growth and development of maize seedlings [7]. Abnormal changes in nitrogen metabolizing enzymes will likely cause an imbalance in nitrogen metabolism [37]. The present study showed that NR, GS, and GOGAT enzyme activities were reduced and GDH activity was enhanced in both shoots and roots of maize seedlings during Mn deficiency. Among them, the decrease in nitrogen-metabolizing enzyme activities likely resulted from the Mn deficiency severely inhibiting photosynthesis in maize seedlings. This resulted in the suppression of the normal metabolic cycle, as carbon metabolism could not provide the seedlings with a sufficient carbon skeleton, thereby reducing their energy. This result was similar to the results of a previous study [25]. Our research indicates that the significantly increased GDH activity possibly resulted from the ammonium assimilation during Mn deficiency having relied mainly on the GDH pathway rather than the GS–GOGAT cycle. Moreover, the tolerant genotype maintained higher nitrogen-metabolizing enzyme activity than the sensitive genotype during Mn deficiency, and an Mn-efficient genotype maintained a higher NR activity than an inefficient genotype during Mn deficiency in rice leaves [16]. In this study, the shoot dry weight, plant height, leaf area, and chlorophyll content of the sensitive genotype were highly significantly and positively correlated with NR, GS, and GOGAT activities, indicating that the growth of maize seedlings was affected by the significant reduction in nitrogen-metabolizing enzyme activities in the sensitive genotype during Mn deficiency stress. The tolerant genotype has a high capacity for efficient N utilization during Mn deficiency stress, thus promoting crop growth and development, quality, and yields.

Previous studies have indicated that key activities of nitrogen metabolism affected the conversion and utilization of nitrogen to plants [37,41]. Manganese deficiency was also found to result in reduced total nitrogen and protein content in studies on chickpea, maize leaves, and sugarcane [11,20,25]. Our study demonstrates that the sensitive genotype had a greater decrease in total nitrogen content, which indicates that Mn deficiency stress inhibited its N-absorbing ability more severely. Moreover, we observed that as opposed to the tolerant genotype, Mn deficiency led to an increase in free amino acid content in the sensitive genotype, and the soluble protein content of the leaves increased first before decreasing. This may be related to the osmoregulation of the plant system, which is consistent with the results of previous studies [42,43]. During mild Mn deficiency, nitrogen assimilation may have been inhibited, resulting in a significant decrease in translocation to the above-ground and partial accumulation of nitrate nitrogen in the roots. The decrease in nitrate nitrogen contents of both roots and leaves in complete Mn deficiency may have resulted from the inhibition in nitrogen uptake by the root system. In summary, these findings suggested that the significant decrease in the activity of the sensitive genotypes NR, GS, and GOGAT under Mn deficiency was a key factor in the significant reduction in dry weight, plant height, leaf area, and chlorophyll content of maize seedlings, which severely inhibited their growth and development. In addition, the activities of key enzymes of nitrogen metabolism and the accumulation of nitrogenous material were relatively less inhibited in the tolerant genotypes.

### 3.3. Effect of Mn Deficiency on C/N and R/S of Maize Seedlings of Different Genotypes

C/N reflects the relative intensity and coordination of C and N metabolism in plants [7]. Carbon metabolism and nitrogen metabolism are important metabolic pathways in plants. Nitrogen assimilation requires reduced ferredoxin, ATP, and NADH produced by photoreaction, as well as the synthesis of amino acids using keto acids synthesized by carbon metabolism as carbon skeletons (Figure 9). Concurrently, carbon metabolism requires nitrogen assimilation to provide the necessary organic substances such as enzymes and proteins [38]; for example, Rubisco activity and chlorophyll content are closely related to nitrogen assimilation [36]. In this study, the C/N of the tolerant genotype increased significantly during Mn deficiency, whereas that of the sensitive genotype showed an increase and then a decrease; the root C/N showed a significant increase, and the sensitive genotype presented a greater degree of increase. The tolerant genotype always maintained a higher level of carbon metabolism during Mn deficiency stress with relatively small changes. This indicated that the tolerant genotype has a stronger ability to balance the carbon and nitrogen metabolism of plants during Mn deficiency than the sensitive genotypes and has a better resistance to Mn-deficiency stress.

Manganese deficiency severely affected the carbon and nitrogen metabolism capacity of maize seedlings, leading to a decrease in dry weight both above and below ground. The increase in R/S of the sensitive genotype is likely related to the transportation and distribution of the photosynthetic assimilates. The significant accumulation of soluble sugar content in the root system of maize seedlings of sensitive genotypes Mo17 during Mn deficiency indicated that photosynthetic assimilates produced by leaves were preferentially supplied to the root system to enhance water and nutrient uptake in order to promote root growth, which is consistent with the findings of a previous study [19]. Furthermore, studies in wheat demonstrated that reduced aboveground NRAMP6 protein levels during Mn deficiency preferentially maintained root growth [14]. However, it was likely that the Mn deficiency contributed to a significant increase in root free amino acid content, thereby improving osmoregulation and promoting root growth [42,43]. The tolerant genotype showed relatively little damage to its leaves and roots; the transportation and distribution of the assimilates were not significantly affected, and no significant changes in R/S were observed. In conclusion, the decrease in shoot and root dry weight as well as the increase in R/S in the sensitive genotype was higher than those in the tolerant genotype during Mn-deficiency stress.

## 4. Materials and Methods

### 4.1. Plant Materials

The genotypes of the test materials are as follows: maize inbred line B73 is a Mn-deficiency tolerant genotype, and maize inbred line Mo17 is a Mn-deficiency sensitive genotype [10]. In this study, B73 and Mo17 and their hybrids B73 × Mo17 were selected as the test materials.

### 4.2. Experimental Design

This experiment was conducted from May to September 2022 in the laboratory of the Agronomy Building of Northeastern Agricultural University, Harbin, Heilongjiang Province. A two-way randomized block design hydroponic experiment was used to select three maize materials, B73, Mo17, and B73 × Mo17, as factor A and four MnSO_4_ concentrations of 0.00 (0%), 2.23 (10%), 11.65 (50%), and 22.30 mg/L (100%, control [19]), as factor B. There were 4 treatments (MnSO_4_ concentrations) in the experiment, and each treatment was replicated thrice.

Seeds of maize B73, Mo17, and B73 × Mo17 were disinfected in 2% sodium hypochlorite solution for 8 min to kill fungal spores on the seed surface, rinsed thoroughly with deionized water, and soaked in deionized water for 6 h. The seeds were placed in Petri dishes with two layers of moist filter paper and incubated in the dark at 25 °C for 3 days. The seeds were rinsed daily, left to transplant into large trays after 3 days, and watered daily. The endosperm was removed at the two-leaf and one-heart stage of the maize seedlings. The uniformly growing seedlings were then selected and transplanted into large 1 L black beakers containing 11.65 mg/L MnSO_4_-H_2_O Hoagland nutrient solution for 5 days, with 7 seedlings per pot. Subsequently, the maize seedlings were switched to the full nutrient solution culture, and the Mn deficiency treatment was started. The basic formula of the Hoagland nutrient solution is as follows: Ca(NO_3_)_2_ 945 mg/L, KNO_3_ 506 mg/L, NH_4_NO_3_ 80 mg/L, KH_2_PO_4_ 136 mg/L, and MgSO_4_·7H_2_O 493 mg/L. Iron salt solutions include: FeSO_4_·7H_2_O 2.78 g, EDTA-2Na 3.73 g, and H_2_O 500 mL. The trace element liquid includes: KI 0.83 mg/l, H_3_BO_3_ 6.2 mg/L, MnSO_4_ 22.3 mg/L, ZnSO_4_·7H_2_O 8.6 mg/L, Na_2_MoO_4_ 0.25 mg/L, CuSO_4_ 0.025 mg/L, and CoCl_2_·6H_2_O 0.025 mg/L. Four MnSO_4_ concentration gradients were set at 0%, 10%, 50%, and 100% (CK), and all nutrient solutions were the same except for the varying Mn concentrations. The pH of the nutrient solution was adjusted to 6.0 ± 0.1 with NaOH. The nutrient solution was renewed every 3 days with continuous aeration for 20 min every 4 h. Plants were grown at a diurnal temperature of 25/22 °C and photoperiod regimes of 14/10 h (light/dark). Culture pots were arranged randomly, and their positions changed after each nutrient solution change. After 16 days of Mn deficiency treatment, the most recently expanded leaves and roots were sampled, and physiological indicators were measured. Three biological replicates of each treatment were used.

### 4.3. Determination of Indicators

#### 4.3.1. Determination of Plant Growth

After 16 days of the Mn deficiency treatment, the plant height (the vertical distance from the leaf tip to the base was the height of maize seedlings [44]), leaf length, and leaf width were measured with a tape measure, and the leaf area calculated (leaf area = 0.75 × leaf length × leaf width, 0.75 is a constant [45]). The shoot and root dry weights of maize seedlings were measured using an electronic analytical balance (AEL-160-21, Shimadzu Corporation, Kyoto, Japan). Root system images were obtained using a root scanner (Microtek ScanMaker i 800 Plus, Shanghai Zhongjing Technology Co., Ltd., Shanghai, China); root length, surface area, volume, and mean diameter were analyzed using the LS-A root analysis system (Hangzhou Wanshen Test Technology Co., Ltd., Hangzhou, China). Root vigor was determined using the triphenyl tetrazolium chloride (TTC) method [46]. Samples were dried in an oven (DHG-9140A, Shanghai Yiheng Scientific Instrument Co., Ltd., Shanghai, China) at 105 °C for 30 min and further dried while maintained at constant weight at 80 °C, and their dry weights (DW) were determined.

#### 4.3.2. Determination of Chlorophyll Content and Carotenoid Content

An amount of 0.1 g of fresh leaf samples were ground in 2 mL of 95% ethanol, then rinsed in the mortar with 8 mL of 95% ethanol and transferred to 10 mL centrifuge tubes for mixing. The homogenate was soaked overnight at 4 °C in the dark. After filtration, the supernatant was collected, and the absorbance was measured at 470 nm, 649 nm, and 665 nm using a spectrophotometer (UV-5500, Shanghai Chemical Laboratory Equipment Co., Ltd., Beijing, China). The contents of chlorophyll a (Chl a), chlorophyll b (Chl b), total chlorophyll (Chl a+b), and carotenoids (Car) were calculated according to the equations reported by Xiong et al. [47] and Polishchuk and Antonyak [48], with three replications for each treatment.

#### 4.3.3. Gas Exchange Parameters Determination

The net photosynthetic rate (Pn, μmol·m^−2^ s^−1^), stomatal conductance (Gs, μmol·m^−2^·s^−1^), intercellular CO_2_ concentration (Ci, μmol·m^−2^ s^−1^), and transpiration rate (Tr, μmol·m^−2^·s^−1^) of the latest expanded leaves of maize seedlings were measured using a Yaxin-1101 photosynthesis tester from 9:00 to 11:30 a.m.

#### 4.3.4. Determination of Chlorophyll Fluorescence Parameters

Maize seedling chlorophyll fluorescence parameters were measured using a chlorophyll fluorometer (Junior-PAM, Heinz Walz GmbH, Effeltrich, Germany) on the middle part of the latest fully expanded leaves after a 30 min dark treatment. These parameters were the maximum photochemical efficiency Fv/Fm of PSII, the relative electron transfer rate ETR, the photochemical quenching coefficient qP, the non-photochemical burst parameter NPQ, the actual quantum yield Y(II) of PSII, the quantum yield Y(NPQ) of non-photochemical quenching, and Y(NO).

#### 4.3.5. Determination of Nonstructural Carbohydrate Content and Enzyme Activities Related to Carbon Metabolism

The total soluble sugar content was determined from fresh samples using the anthrone sulfate method [49], and the starch content was determined using the anthrone method [50]. The sucrose content was determined from dry samples using the resorcinol method [51], where nonstructural carbohydrate content (NSC) is the sum of the soluble sugar and starch contents [52]. The glucose and fructose contents as well as neutral invertase (NI) were determined using kits G0504F, G0505F, and G0516W purchased from Grace Biotechnology Company (Suzhou, China, www.geruisi-bio.com). Sucrose synthase (synthesis direction) (SS) and SPS activities were measured using kits A097-1-1 and A098-1-1 purchased from the Nanjing Jiancheng Institute of Biological Engineering Co., Ltd. (China, www.njjcbio.com). All physiological parameters measured with the kits were performed using fresh samples, and the procedure was strictly based on the manufacturer’s instructions.

#### 4.3.6. Evaluation of Nitrogen Metabolism-Related Product Content and Key Enzyme Activities

The total nitrogen content was determined from dry samples via concentrated sulfuric acid-hydrogen peroxide digestion and semi-micro Kjeldahl nitrogen determination [53]. The nitrate content in fresh samples was determined using the salicylic acid method [54], and the free amino acid content was determined via the ninhydrin staining method [55]. The soluble protein content from fresh samples was determined using the Coomassie Brilliant Blue G-250 solution [56]. The nitrate reductase and glutamate synthase activities of leaves and roots were determined via kits from Grace Biotechnology Company (Suzhou, China) for (NR, G0402F), leaf glutamate synthase (Fd-GOGAT, G0404W96), and root glutamate synthase (NADH-GOGAT, G0403W). Leaf and root GS and GDH activities were measured using kits A047-1-1 and A125-1-1 purchased from Nanjing Jiancheng. All physiological parameters measured with the kits were performed using fresh samples, and the procedure was performed strictly according to the manufacturer’s instructions. C/N = (soluble sugars content + starch content)/(soluble protein content + free amino acid content).

#### 4.3.7. Statistical Analysis

Data were statistically analyzed using Microsoft Excel 2007 and SPSS 17 software. Two-way ANOVA with post hoc LSD tests were used to compare differences between treatments at the significance level of *p* < 0.05. Correlation analysis was performed using Origin Pro 2022b.

## 5. Conclusions

Mn deficiency negatively affected photosynthetic gas exchange parameters, chlorophyll fluorescence parameters, and chlorophyll content in different genotypes of maize seedlings. This resulted in a reduced ability of maize seedling leaves to convert photosynthetic energy into chemical energy, reductions in the electron transfer rate and heat dissipation, and an inability to provide sufficient reducing power and energy for carbon and nitrogen metabolism. Mn deficiency further inhibited NR, GS, and GOGAT activities in the leaves and roots of maize seedlings, which caused reduced nitrogen uptake by the roots and significantly decreased protein synthesis in the leaves and roots; unsurprisingly, the nitrogen metabolism capacity of maize seedlings decreased severely. However, maize seedlings resisted Mn-deficiency stress by enhancing soluble carbohydrate accumulation. Compared with the sensitive genotype, the tolerant genotype was less damaged and improved its osmoregulation ability by effectively enhancing SPS and SS enzyme activities, accumulating more soluble carbohydrates and maintaining a relatively high level of carbon metabolism. This further alleviated the overall damage caused by Mn deficiency and effectively improved the metabolic capacity for maize seedling growth.

## Figures and Tables

**Table 1:** Analysis of variance of Mn deficiency on dry weight and plant height of maize seedlings of different genotypes.

Source of Variation	Degree of Freedom	Shoot Dry Weight		Root Dry Weight		Plant Height	
		Mean Square	*p*	Mean Square	*p*	Mean Square	*p*
A	2	0.0569	0.0001	0.0036	0.0001	720.0411	0.0001
B	3	0.0511	0.0001	0.0010	0.0001	212.2933	0.0001
A × B	6	0.0061	0.0001	0.0000	0.3776	28.3233	0.0017

A: Genotypes, B: MnSO_4_ concentrations, A × B: Genotypes × Mn concentrations interactions.

**Table 2:** Effect of Mn deficiency on the dry weight and morphological phenotypes of maize seedlings.

Genotypes	MnSO_4_ Concentrations	Shoot Dry Weight g/Plant	Root Dry Weight g/Plant	Root to Shoot Ratio	Plant Height cm/Plant	Leaf Area cm^2^/Plant
Mo17	0%	0.249 ± 0.019 Cc	0.067 ± 0.002 Bb	0.271 ± 0.012 Aa	44.8 ± 1.2 Bc	124.8 ± 5.1 Bc
	10%	0.293 ± 0.022 Cc	0.069 ± 0.002 Bb	0.236 ± 0.012 Ab	48.6 ± 2.4 Bc	136.6 ± 15.0 Bbc
	50%	0.445 ± 0.022 Bb	0.086 ± 0.005 Ba	0.193 ± 0.004 Ac	54.4 ± 2.3 Bb	160.2 ± 9.7 Bb
	100%	0.511 ± 0.018 Ba	0.093 ± 0.005 Ba	0.182 ± 0.004 Ac	62.7 ± 1.5 Ba	200.2 ± 13.5 Ba
B73	0%	0.363 ± 0.016 Bb	0.055 ± 0.004 Cb	0.151 ± 0.005 Cab	48.5 ± 1.2 Bb	125.5 ± 14.5 Bb
	10%	0.415 ± 0.024 Ba	0.060 ± 0.005 Bab	0.145 ± 0.005 Cb	50.8 ± 1.7 Bab	151.8 ± 7.2 Ba
	50%	0.438 ± 0.016 Ba	0.070 ± 0.003 Ca	0.159 ± 0.002 Ba	52.3 ± 1.8 Bab	158.4 ± 15.8 Ba
	100%	0.458 ± 0.022 Ca	0.068 ± 0.003 Ca	0.149 ± 0.003 Bab	55.7 ± 2.3 Ca	170.3 ± 6.7 Ca
B73 × Mo17	0%	0.440 ± 0.024 Ab	0.086 ± 0.005 Ab	0.195 ± 0.004 Ba	61.7 ± 1.0 Ab	200.4 ± 10.9 Ac
	10%	0.469 ± 0.024 Ab	0.090 ± 0.008 Ab	0.191 ± 0.008 Ba	62.1 ± 2.8 Ab	213.9 ± 11.9 Abc
	50%	0.557 ± 0.020 Aa	0.109 ± 0.007 Aa	0.195 ± 0.005 Aa	70.1 ± 1.7 Aa	243.9 ± 21.8 Aa
	100%	0.594 ± 0.024 Aa	0.107 ± 0.005 Aa	0.187 ± 0.009 Aa	69.2 ± 2.5 Aa	235.5 ± 15.1 Aab

According to the LSD test different capital letters: significant difference between genotypes (*p* < 0.05); different lowercase letters: significant difference between MnSO_4_ treatments (*p* < 0.05). Data represent the mean ± standard deviation (*n* = 3).

**Table 3:** Analysis of variance of Mn deficiency on root length of maize seedlings of different genotypes.

Source of Variation	Sum of Squares	Degree of Freedom	Mean Square	F	*p*
A	486,005.9245	2	243,002.9622	123.9268	0.0001
B	1,493,706.237	3	497,902.0789	253.9203	0.0001
A × B	684,510.4861	6	114,085.081	58.1812	0.0001

A: Genotypes, B: MnSO_4_ concentrations, A × B: Genotypes × Mn concentrations.

**Table 4:** Effect of Mn deficiency on root morphological phenotype and root activity of maize seedlings.

Genotypes	MnSO_4_ Concentrations	Total Root Length cm/Plant	Root Surface Area cm^2^/Plant	Root Volume cm^3^/Plant	Root Average Diameter/mm	Root Activity /TTF μg·g^−1^·h^−1^
Mo17	0%	340.14 ± 26.63 Bc	61.64 ± 14.73 Ac	1.84 ± 0.38 Bd	0.65 ± 0.01 Aa	81.25 ± 3.16 Ac
	10%	398.50 ± 25.93 Bbc	76.65 ± 8.82 Abc	3.04 ± 0.46 Ac	0.65 ± 0.08 Aa	99.68 ± 4.01 Ab
	50%	529.61 ± 27.65 Cb	105.67 ± 10.38 Bb	5.23 ± 0.71 Bb	0.68 ± 0.05 Aa	110.11 ± 10.00 Ab
	100%	1190.95 ± 25.42 Aa	218.45 ± 12.78 Aa	8.11 ± 0.84 Aa	0.62 ± 0.01 Ba	163.49 ± 12.01 Aa
B73	0%	506.73 ± 22.28 Ab	77.95 ± 4.24 Ab	2.40 ± 0.02 ABb	0.49 ± 0.02 Bb	51.67 ± 3.79 Bb
	10%	528.76 ± 22.06 ABb	77.99 ± 3.08 Ab	2.46 ± 0.10 Ab	0.49 ± 0.01 Bb	52.88 ± 1.37 Bb
	50%	714.81 ± 69.28 Ba	124.24 ± 14.28 Ba	4.39 ± 0.01 Ba	0.58 ± 0.01 Ba	77.44 ± 9.11 Ba
	100%	726.29 ± 28.13 Ba	118.71 ± 12.95 Ba	4.17 ± 0.04 Ba	0.57 ± 0.02 Ba	89.25 ± 2.27 Ba
B73 × Mo17	0%	647.28 ± 30.24 Ab	95.50 ± 7.86 Ab	3.08 ± 0.46 Ab	0.52 ± 0.01 Bc	38.45 ± 0.91 Cb
	10%	615.53 ± 39.45 Ab	81.10 ± 5.23 Ab	2.38 ± 0.38 Ab	0.49 ± 0.02 Bc	43.11 ± 6.54 Bb
	50%	1156.15 ± 40.92 Aa	251.64 ± 9.57 Aa	9.49 ± 0.58 Aa	0.63 ± 0.01 ABb	49.47 ± 2.94 Cb
	100%	1034.73 ± 47.35 Aa	223.71 ± 2.04 Aa	8.76 ± 0.67 Aa	0.76 ± 0.02 Aa	69.71 ± 9.07 Ca

According to the LSD test different capital letters: significant difference between genotypes (*p* < 0.05); different lowercase letters: significant difference between MnSO_4_ treatments (*p* < 0.05). Data represent the mean ± standard deviation (*n* = 3).

## Data Availability

The data presented in this study are available within the article.
